# POD24的脾边缘区淋巴瘤的临床特征及预后

**DOI:** 10.3760/cma.j.cn121090-20240729-00285

**Published:** 2025-01

**Authors:** 露 王, 晴 施, 维莅 赵, 黎 王

**Affiliations:** 1 上海血液学研究所，组学与疾病国家重点实验室，国家转化医学研究中心，上海交通大学医学院附属瑞金医院血液科，上海 200025 Shanghai Institute of Hematology, State Key Laboratory of Genomics and Disease, National Translational Medicine Research Center, Department of Hematology, Ruijin Hospital, Shanghai Jiaotong University School of Medicine, Shanghai 200025, China; 2 太原钢铁（集团）有限公司总医院（山西医科大学第六医院、山西医科大学第六临床医学院）血液科，太原 030000 Department of Hematology, General Hospital of Taiyuan Iron and Steel（Group）Company Limited（The Sixth Hospital of Shanxi Medical University, The Sixth Clinical Medical College of Shanxi Medical University）, Taiyuan 030000, China

**Keywords:** 淋巴瘤，B细胞，边缘区, 脾肿瘤, 24个月内疾病进展, 预后, Lymphoma, B-cell, marginal zone, Splenic neoplasms, Disease progression within 24 months, Prognosis

## Abstract

**目的:**

探讨脾边缘区淋巴瘤（splenic marginal zone lymphoma，SMZL）确诊后24个月内疾病进展（POD24）患者的临床特征、预后及危险因素。

**方法:**

回顾性分析2009年12月至2022年10月在上海交通大学医学院附属瑞金医院就诊的88例初治SMZL患者的临床资料，根据POD24发生情况分组并进行预后评估及临床特征比较。

**结果:**

男45例（51.1％），女43例（48.9％），诊断时中位年龄59（24～82）岁。10例（11.4％）发生POD24。POD24组总生存（OS）期和无进展生存（PFS）期短于非POD24组［中位OS期：77（11～159）个月对未达到，*P*<0.001；中位PFS期：15（4～24）个月对121（24～154）个月，*P*<0.001］。单因素Cox分析显示，美国东部肿瘤协作组（ECOG）评分≥2分［*HR*＝8.942（95％ *CI* 1.097～72.910），*P*＝0.041］、年龄调整的国际预后指数（aaIPI）评分高危［*HR*＝5.070（95％ *CI* 1.256～20.461），*P*＝0.023］、POD24［*HR*＝14.049（95％ *CI* 3.339～59.107），*P*<0.001］、发生组织转化［*HR*＝7.819（95％ *CI* 1.952～31.316），*P*＝0.004］、初始治疗后疾病未缓解［*HR*＝6.080（95％ *CI* 1.439～25.690），*P*＝0.014］为SMZL患者OS的影响因素。多因素分析显示，POD24［*HR*＝5.859（95％ *CI* 1.249～27.475），*P*＝0.025］、发生组织转化［*HR*＝5.520（95％ *CI* 1.050～29.009），*P*＝0.044］为OS的独立预后不良因素。单因素Logistic分析显示，ECOG评分≥2分［*HR*＝7.556（95％ *CI* 1.498～38.110），*P*＝0.014］、aaIPI评分高危［*HR*＝5.500（95％ *CI* 1.378～21.945），*P*＝0.016］、发生组织转化［*HR*＝8.000（95％ *CI* 1.759～36.383），*P*＝0.007］、初始治疗后疾病未缓解［*HR*＝9.136（95％ *CI* 2.216～37.675），*P*＝0.002］与POD24的发生相关。多因素分析显示，初始治疗后疾病未缓解［*HR*＝8.253（95％ *CI* 1.681～40.518），*P*＝0.009］为影响POD24的独立危险因素。

**结论:**

POD24、发生组织转化为影响SMZL患者OS的独立预后不良因素。POD24患者发生组织转化风险高。初始治疗后疾病未缓解为影响POD24患者的独立危险因素。

## Abstract (en)

**Objective:**

To investigate the clinical characteristics, prognosis, and risk factors associated with disease progression within 24 months（POD24）after diagnosis in patients with splenic marginal zone lymphoma（SMZL）.

**Methods:**

Clinical data from 88 newly diagnosed SMZL patients treated at Ruijin Hospital, Shanghai Jiaotong University School of Medicine, between December 2009 and October 2022, were retrospectively analyzed. Patients were grouped based on the presence of POD24 for prognostic evaluation and comparison of clinical features.

**Results:**

There were 45 males（51.1％）and 43 females（48.9％）, with a median age of 59（24–82）years at the time of diagnosis. Ten（11.4％）cases occurred POD24. The overall survival（OS）time and progression-free survival（PFS）time in the POD24 group were shorten than non-POD24 group［median OS time: 77（11–159）months *vs* not reached, *P*<0.001; 15（4–24）months *vs* 121（24–154）months, *P*<0.001］. Univariate Cox analysis showed that Eastern Cooperative Oncology Group（ECOG）score ≥ 2［*HR*＝8.942（95％ *CI* 1.097–72.910）, *P*＝0.041］, age-adjusted International Prognostic Index（aaIPI）score of high-risk［*HR*＝5.070（95％ *CI* 1.256–20.461）, *P*＝0.023］, POD24［*HR*＝14.049（95％ *CI* 3.339–59.107）, *P*<0.001］, occurrence of tissue transformation［*HR*＝7.819（95％ *CI* 1.952–31.316）, *P*＝0.004］, and disease unremission status after initial treatment［*HR*＝6.080（95％ *CI* 1.439–25.690）, *P*＝0.014］were the influencing factors for OS in SMZL patients. Multivariate analysis showed that POD24［*HR*＝5.859（95％ *CI* 1.249–27.475）, *P*＝0.025］and occurrence of tissue transformation［*HR*＝5.520（95％ *CI* 1.050–29.009）, *P*＝0.044］were independent prognostic factors affecting OS. Univariate logistic analysis showed that ECOG ≥ 2［*HR*＝7.556（95％ *CI* 1.498–38.110）, *P*＝0.014］, high risk of aaIPI score［*HR*＝5.500（95％ *CI* 1.378– 21.945）, *P*＝0.016］, occurrence of tissue transformation［*HR*＝8.000（95％ *CI* 1.759–36.383）, *P*＝0.007］, and disease unremission status after initial treatment［*HR*＝9.136（95％ *CI* 2.216–37.675）, *P*＝0.002］were the influencing factors of POD24. Multifactorial analysis showed that disease unremission after initial treatment［*HR*＝8.253（95％ *CI* 1.681–40.518）, *P*＝0.009］was an independent risk factor affecting POD24.

**Conclusion:**

POD24 and tissue transformation are independent poor prognostic factors for OS in SMZL patients. Patients with POD24 are at a higher risk of developing tissue transformation. The failure to alleviate the disease after initial treatment is an independent risk factor affecting POD24 patients.

脾边缘区淋巴瘤（splenic marginal zone lymphoma，SMZL）是一类以脾受累为突出表现的惰性B细胞淋巴瘤，仅占淋巴瘤的1.0％～2.7％[1]。根据世界卫生组织（WHO）分类，SMZL属于边缘区淋巴瘤（MZL）中的一种，约占20％[2]。与结外MZL和结内MZL不同，SMZL的特点是脾肿大、骨髓受累，外周血绒毛淋巴细胞存在差异，可能会出现细胞减少症，继发于脾功能亢进、自身免疫现象或骨髓浸润[3]–[5]。SMZL通常表现为慢性过程，中位发病年龄为65岁。尽管SMZL中位生存期较长，但仍存在异质性。目前仍缺少预测SMZL预后的有效指标。国外有研究指出，24个月内疾病进展（POD24）的SMZL患者较非POD24患者预后差[6]。本研究整理并总结了本中心SMZL患者的资料，分析POD24对SMZL患者总生存（OS）的影响及发生POD24患者的临床特征，以期及早识别高危患者，进行个体化治疗，从而改善SMZL患者的预后。

## 病例与方法

1. 病例：回顾性分析2009年12月至2022年10月在上海交通大学医学院附属瑞金医院就诊的88例初治SMZL患者的临床资料。诊断标准参照脾B细胞淋巴瘤协作组（SBLG）最低诊断标准[7]，需满足：脾组织学表现（WHO标准[8]）+慢性淋巴细胞白血病（CLL）免疫表型积分≤2分；或无法获得脾组织时，表现为典型血液和骨髓形态学+免疫表型+窦内CD20阳性细胞浸润。

2. 方法：收集患者的临床资料，包括性别、年龄、B症状（发热、盗汗、体重减轻）、美国东部肿瘤协作组（ECOG）评分、疾病分期（Ann Arbor分期）、HGB、PLT、血清白蛋白（ALB）、LDH、β_2_微球蛋白（β_2_-MG）、可溶性白细胞介素-2受体（sIL-2R）、治疗方案、治疗期间发生组织转化情况等。采用美国国家综合癌症网络国际预后指数（NCCN-IPI）、黏膜相关淋巴组织国际预后指数（MALT-IPI）、边缘区淋巴瘤国际预后指数（MZL-IPI）、国际预后指数（IPI）、年龄调整的IPI（aaIPI）分别对患者进行危险度分层。

3. 治疗方案：88例患者中，4例进行了单纯脾切除治疗；1例中药治疗；83例患者接受包括R-CHOP（利妥昔单抗+环磷酰胺+阿霉素+长春新碱+泼尼松）方案、R-COP（利妥昔单抗+环磷酰胺+长春新碱+泼尼松）方案、CHOP（环磷酰胺+阿霉素+长春新碱+泼尼松）方案、BR（苯达莫司汀+利妥昔单抗）方案、GB（奥妥珠单抗+苯达莫司汀）方案、R2（利妥昔单抗+来那度胺）方案在内的化疗，其中46例行单纯化疗，37例行脾切除联合化疗。

4. 疗效评价：根据SBLG的疗效标准[7]：①脾切除术：有效：血细胞计数≥50％，无淋巴细胞进行性增多，并且在骨髓浸润程度上无变化。②非手术治疗：完全缓解（CR）：器官肿大恢复正常，血细胞计数正常（HGB>120 g/L、PLT>100×10^9^/L、ANC>1.5×10^9^/L），并且外周血中无克隆性B细胞，免疫组化在骨髓中未检测到微小浸润；部分缓解（PR）：疾病症状改善≥50％，包括脾体积缩小或恢复正常，血细胞减少或改善，并且原有淋巴结缩小或恢复正常，骨髓中淋巴细胞数量减少且部分造血功能恢复；治疗有效包括上述CR及PR；无反应：疾病症状改善10％；疾病进展：以上症状发生恶化。

5. 随访：采用查阅门诊及住院病历和电话随访患者，随访截止时间为2024年2月29日。无进展生存（PFS）期定义为自诊断至疾病发生进展的时间。OS期定义为自诊断至死亡或随访结束的时间。POD24指确诊后24个月内出现疾病进展。

6. 统计学处理：使用SPSS27.0软件进行统计学分析，计量资料采用*M*（范围）表示，计数资料采用例数（％）进行表示，比较用卡方检验或Fisher精确概率法，采用Kaplan-Meier曲线进行预后分析，并行Log-rank检验。利用Cox分析探究POD24与SMZL预后的相关性。运用Logistics分析探究POD24的危险因素。*P*<0.05为差异有统计学意义。

## 结果

1. 临床特征：88例患者中，男45例（51.1％），女43例（48.9％），诊断时中位年龄59（24～82）岁。>60岁42例（47.7％）、≤60岁46例（52.3％），96.6％的患者有骨髓受累，所有患者均有脾受累，Ann Arbor分期均为Ⅲ/Ⅳ期，伴有B症状30例（38.5％），35例（44.9％）患者初诊时ECOG评分≥2分。50例患者行靶向基因测序，29例（58％）检出基因突变：MYD88突变8例（27.6％）、CXCR4突变5例（17.2％）、TNFAIP3突变4例（13.8％）、TP53突变3例（10.3％）、BIRC3突变3例（10.3％）、KMT2D突变3例（10.3％）、IRF8突变2例（6.9％）、TET2突变2例（6.9％）、SPEN突变2例（6.9％）、CREBBP突变2例（6.9％）。

POD24组与非POD24组SMZL临床特征比较：见表1，诊断24个月内10例（11.4％）患者发生疾病进展，另外78例患者被归为非POD24组。POD24组较非POD24组初诊时ECOG评分≥2分比例（80.0％对34.6％，*P*＝0.012）、sIL-2R>710 U/ml的比例明显增高（90.0％对56.4％，*P*＝0.047）。而B症状、HGB<120 g/L、PLT<100×10^9^/L及ALB<35 g/L的比例在POD24组中也较高，但与非POD24组的差异均无统计学意义（均*P*>0.05）。POD24组较非POD24组初诊时aaIPI评分高危的比例明显增高（50.0％对15.4％，*P*＝0.009）；而初诊时IPI评分高危、NCCN-IPI评分高危、MZL-IPI评分高危的比例在POD24组中也高于非POD24组，但差异均无统计学意义（均*P*>0.05）。10例POD24患者中仅有3例行靶向基因测序，1例阴性、1例MYD88突变（33.3％）和1例BCOR突变（33.3％）。

**表1:** POD24组与非POD24组脾边缘区淋巴瘤患者初诊时的临床特征比较［例（％）］

特征	POD24（10例）	非POD24（78例）	*χ*^2^值	*P*值
男性	3（30.0）	42（53.8）	2.017	0.191
年龄>60岁	5（50.0）	37（47.4）	0.023	0.879
B症状	5（50.0）	25（32.1）	1.271	0.260
ECOG评分≥2分	8（80.0）	27（34.6）	5.845	0.012
HGB<120 g/L	8（80.0）	49（62.8）	0.517	0.484
PLT<100×10^9^/L	7（70.0）	29（37.2）	3.950	0.084
ALB<35 g/L	6（60.0）	21（26.9）	4.560	0.062
LDH>192 U/L	5（50.0）	45（57.7）	0.214	0.644
β_2_微球蛋白>2 366 ng/ml	8（80.0）	61（78.2）	0.017	1.000
sIL-2R>710 U/ml	9（90.0）	44（56.4）	4.939	0.047
IPI评分（高危）	5（50.0）	28（35.9）	0.752	0.386
aaIPI评分（高危）	5（50.0）	12（15.4）	6.814	0.009
NCCN-IPI评分（高危）	3（30.0）	8（10.3）	3.159	0.104
MALT-IPI评分（高危）	5（50.0）	49（62.8）	0.614	0.433
MZL-IPI评分（高危）	4（40.0）	16（20.5）	1.917	0.226

**注** POD24：24个月内疾病进展；B症状：发热、盗汗、体重减轻；ECOG：美国东部肿瘤协作组；ALB：血清白蛋白；sIL-2R：可溶性白细胞介素-2受体；IPI：国际预后指数；aaIPI：年龄调整的国际预后指数；NCCN-IPI：美国国家综合癌症网络国际预后指数；MALT-IPI：黏膜相关淋巴组织国际预后指数；MZL-IPI：边缘区淋巴瘤国际预后指数

2. 生存分析：88例患者中，31例出现疾病进展，8例死亡，中位随访时间为55.5（11～162）个月，患者总体中位OS期未达到，2年OS率为97.7％、5年OS率为94.3％、10年OS率为79.7％（图1A）。患者总体中位PFS期为114（4～154）个月，2年PFS率为88.6％、5年PFS率为66.4％、10年PFS率为47.4％（图1B）。

**图1:**
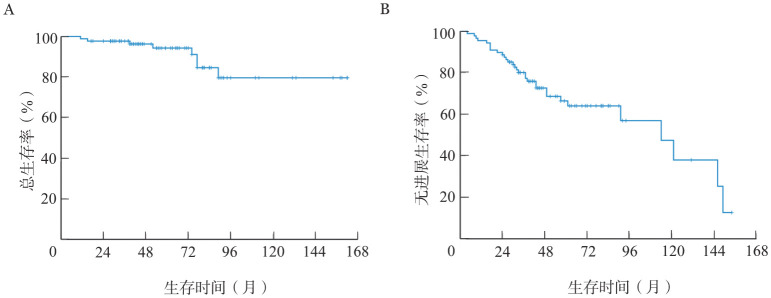
脾边缘区淋巴瘤患者的总生存（A）和无进展生存（B）曲线

3. POD24患者与非POD24患者的预后：如图2所示，POD24组10例患者中有5例（50.0％）死亡，中位OS期和中位PFS期分别为77（11～159）个月和15（4～24）个月，非POD24组中位OS期未达到，中位PFS期为121（24～154）个月。非POD24组中位OS期和中位PFS期均长于POD24组（均*P*<0.001）。POD24组的2年OS率为80.0％、10年OS率为20.0％；非POD24组的2年OS率为100％、10年OS率为92.3％。

**图2:**
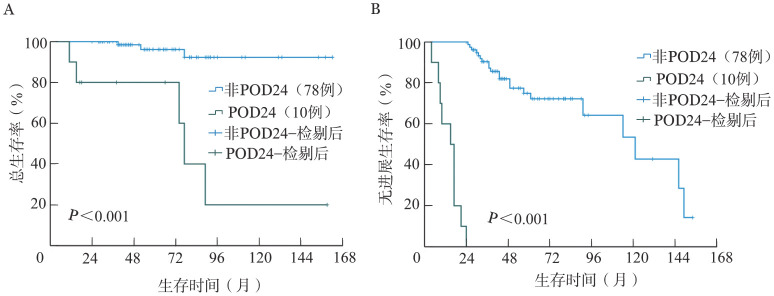
POD24组和非POD24组脾边缘区淋巴瘤患者的总生存（A）和无进展生存（B）曲线

67例（76.1％）患者初始治疗后疾病达缓解（CR+PR），其中POD24组仅4例达缓解。治疗期间10例（11.4％）发生组织转化，中位转化时间为28.5（4～114）个月，8例（9.1％）转化为弥漫大B细胞淋巴瘤（DLBCL），2例（2.3％）转化为滤泡性淋巴瘤（FL），其中POD24组发生组织转化4例，均转化为DLBCL。单因素Cox分析显示，ECOG评分≥2分［*HR*＝8.942（95％ *CI* 1.097～72.91），*P*＝0.041］、aaIPI评分高危［*HR*＝5.070（95％ *CI* 1.256～20.461），*P*＝0.023］、POD24［*HR*＝14.049（95％ *CI* 3.339～59.107），*P*<0.001］、发生组织转化［*HR*＝7.819（95％ *CI* 1.952～31.316），*P*＝0.004］、初始治疗后疾病未缓解［*HR*＝6.080（95％ *CI* 1.439～25.690），*P*＝0.014］为SMZL患者OS的影响因素（表2）。对单因素分析中的因变量（*P*<0.05）进行Cox多因素分析，结果显示，POD24［*HR*＝5.859（95％ *CI* 1.249～27.475），*P*＝0.025］、发生组织转化［*HR*＝5.520（95％ *CI* 1.050～29.009），*P*＝0.044］为影响SMZL患者OS的独立预后不良因素（表3）。

**表2:** 脾边缘区淋巴瘤患者关于总生存的单因素分析

相关因素	*HR*（95％ *CI*）	*P*值
男性	0.819（0.204～3.289）	0.779
年龄>60岁	2.774（0.648～11.872）	0.169
B症状	0.899（0.213～3.801）	0.885
ECOG评分≥2分	8.942（1.097～72.91）	0.041
HGB<120 g/L	1.455（0.291～7.285）	0.648
PLT<100×10^9^/L	0.894（0.214～3.741）	0.848
ALB<35 g/L	3.409（0.813～14.293）	0.094
LDH>192 U/L	0.692（0.172～2.784）	0.604
β_2_微球蛋白>2 366 ng/ml	1.388（0.168～11.443）	0.761
sIL-2R>710 U/ml	4.060（0.783～21.039）	0.095
aaIPI评分（高危）	5.070（1.256～20.461）	0.023
IPI评分（高危）	3.479（0.827～14.627）	0.089
NCCN-IPI评分（高危）	4.213（0.810～21.916）	0.087
MALT-IPI评分（高危）	0.627（0.156～2.513）	0.510
MZL-IPI评分（高危）	0.594（0.073～4.846）	0.627
POD24	14.049（3.339～59.107）	<0.001
发生组织转化	7.819（1.952～31.316）	0.004
初始治疗后疾病未缓解	6.080（1.439～25.690）	0.014

**注** B症状：发热、盗汗、体重减轻；ECOG：美国东部肿瘤协作组；ALB：血清白蛋白；sIL-2R：可溶性白细胞介素-2受体；IPI：国际预后指数；aaIPI：年龄调整的国际预后指数；NCCN-IPI：美国国家综合癌症网络国际预后指数；MALT-IPI：黏膜相关淋巴组织国际预后指数；MZL-IPI：边缘区淋巴瘤国际预后指数；POD24：24个月内疾病进展

**表3:** 脾边缘区淋巴瘤患者关于总生存的多因素分析

相关因素	*HR*（95％ *CI*）	*P*值
ECOG评分≥2分	3.319（0.343～32.096）	0.300
aaIPI评分（高危）	4.240（0.417～43.046）	0.222
POD24	5.859（1.249～27.475）	0.025
发生组织转化	5.520（1.050～29.009）	0.044
初始治疗后疾病未缓解	4.825（0.700～33.255）	0.110

**注** ECOG：美国东部肿瘤协作组；aaIPI：年龄调整的国际预后指数；POD24：24个月内疾病进展

3. POD24的危险因素分析：单因素Logistic回归分析显示，ECOG评分≥2分［*HR*＝7.556（95％ *CI* 1.498～38.110），*P*＝0.014］、aaIPI评分高危［*HR*＝5.500（95％ *CI* 1.378～21.945），*P*＝0.016］、发生组织转化［*HR*＝8.000（95％ *CI* 1.759～36.383），*P*＝0.007］、初始治疗后疾病不缓解［*HR*＝9.136（95％ *CI* 2.216～37.675），*P*＝0.002］为POD24的危险因素。进一步行多因素分析显示，初始治疗后疾病未缓解［*HR*＝8.253（95％ *CI* 1.681～40.518），*P*＝0.009］为影响POD24患者的独立危险因素（表4）。

**表4:** 影响脾边缘区淋巴瘤患者POD24的单因素及多因素分析

相关因素	单因素分析		多因素分析	
	*HR*（95％ *CI*）	*P*值	*HR*（95％ *CI*）	*P*值
ECOG评分≥2分	7.556（1.498～38.110）	0.014	6.465（0.892～46.882）	0.065
aaIPI（高危）	5.500（1.378～21.945）	0.016	1.389（0.219～8.818）	0.727
发生组织转化	8.000（1.759～36.383）	0.007	5.253（0.787～35.067）	0.087
初始治疗后疾病未缓解	9.136（2.216～37.675）	0.002	8.253（1.681～40.518）	0.009

**注** POD24：24个月内疾病进展；ECOG：美国东部肿瘤协作组；aaIPI：年龄调整的国际预后指数

## 讨论

SMZL是一种小B淋巴细胞浸润脾和骨髓的疾病。本研究中88例SMZL患者均有脾受累，96.6％的患者有骨髓受累，与国内报道[9]类似。Casulo等[10]在2015年首先提出POD24可作为筛选FL高危患者早期疾病进展的指标。Luminari等[6]的前瞻性研究中首次在MZL患者中提出了POD24的概念。近年来，POD24在霍奇金淋巴瘤、套细胞淋巴瘤、血管免疫母细胞性T细胞淋巴瘤及非滤泡性惰性淋巴瘤等多项研究中被证明是独立的不良预后因素[11]–[14]。本研究共纳入88例SMZL患者，10年PFS率和OS率分别47.4％和79.7％，11.4％的患者经历了POD24，POD24组中位OS期和中位PFS期分别为77个月和15个月，10年OS率为20.0％，低于非POD24组（92.3％）。多因素分析示，POD24、发生组织转化是SMZL患者OS的独立预后不良因素。Iannitto等[15]在SMZL的研究中发现，报告的死亡病例中至少有50％与淋巴瘤进展或组织转化为高级别非霍奇金淋巴瘤有关。吕瑞等[16]对106例SMZL患者的预后分析研究结果显示，POD24组的5年OS率为51.3％，显著低于非POD24组（85.7％），这与我们的研究结果一致。FIL-NF10研究[6]报道了321例MZL患者中POD24组3年OS率低于非POD24组［53％对88％，*HR*＝19.5（95％ *CI* 8.4～45.4），*P*<0.001］，POD24与OS的关系在结外边缘区淋巴瘤（ENMZL）、SMZL和播散性MZL（Diss-MZL）亚组中进一步得到证实：84例SMZL患者POD24组3年OS率为44％，而非POD24组为95％（*P*<0.001）；59例Diss-MZL患者POD24组3年OS率为33％，非POD24组为93％（*P*<0.001）；146例ENMZL患者POD24组的3年OS率为71％，非POD24组为98％（*P*<0.001）。这提示POD24对SMZL患者的OS有独立预后价值。

本组POD24患者初诊时ECOG评分≥2分、sIL-2R>710 U/ml、aaIPI评分高危的比例均显著高于非POD24组，提示初诊时有上述特征的患者更易发生POD24。IL-2R由活化的T淋巴细胞释放，与B细胞和T细胞的增殖有关，该因子的高浓度与非霍奇金淋巴瘤的预后有关。Nozaki等[17]的研究发现，治疗前sIL-2R水平与新诊断的FL患者较差的预后有明显的剂量依赖关系。在Yoshizato等[18]的研究中发现，诊断时的高水平sIL-2R与较短的PFS期相关（*P*＝0.018），在获得缓解的患者中，sIL-2R明显下降（*P*<0.001），而淋巴瘤进展时，sIL-2R则上升（*P*<0.001），sIL-2R与FL患者诊断时和临床治疗过程中的肿瘤负荷相关，可作为淋巴瘤进展的替代标志物。

本研究发现，在治疗期间发生组织转化、初始治疗后疾病未缓解是POD24的危险因素。针对SMZL报道的转化为DLBCL的风险为8％～19％[19]–[21]。本研究中10例（11.4％）发生组织转化，8例（9.1％）转化为DLBCL，与上述报道相似。POD24组发生组织转化4例，均转化为DLBCL。有关FL的研究表明，早期事件中可能会出现更具攻击性的转化病例[22]。FIL-NF10研究[6]中，在90例疾病进展的患者中发现了7例组织转化的MZL，都被算作POD24病例，组织转化可能在确定早期事件的发生方面发挥作用。

综上所述，POD24可作为SMZL治疗过程中筛选高危患者的重要指标，也可作为SMZL患者OS的独立预后不良因素，具有潜在的临床参考价值。

本研究属于单中心回顾性研究，例数少，可追踪的基因检测数据少，POD24组仅3例进行了靶向测序，因此未将基因突变因素纳入预后分析，未来还需要更大规模的病例来验证与POD24相关的基因突变谱，以期更早识别高危及不良预后患者，并开发更有效的治疗靶点，进行个体化治疗，改善SMZL患者的预后。

## References

[1] Cerhan JR, Habermann TM (2021). Epidemiology of marginal zone lymphoma[J]. Ann Lymphoma.

[2] Piris MA, Onaindía A, Mollejo M (2017). Splenic marginal zone lymphoma[J]. Best Pract Res Clin Haematol.

[3] 杨 申淼, 江 倩, 江 滨 (2013). 伴血象异常的脾边缘区淋巴瘤的临床特点[J]. 中国实验血液学杂志.

[4] Sena Teixeira Mendes L, Du MQ, Matutes E (2014). Splenic marginal zone lymphoma: a review of the clinical presentation, pathology, molecular biology, and management[J]. Blood and Lymphatic Cancer: Targets and Therapy.

[5] Kalpadakis C, Pangalis GA, Vassilakopoulos TP (2014). Treatment of splenic marginal zone lymphoma: should splenectomy be abandoned?[J]. Leuk Lymphoma.

[6] Luminari S, Merli M, Rattotti S (2019). Early progression as a predictor of survival in marginal zone lymphomas: an analysis from the FIL-NF10 study[J]. Blood.

[7] Matutes E, Oscier D, Montalban C (2008). Splenic marginal zone lymphoma proposals for a revision of diagnostic, staging and therapeutic criteria[J]. Leukemia.

[8] SH S (2008). Splenic B-cell marginal zone lymphoma[J]. World Health Organization classification of tumours of haematopoietic and lymphoid tissues.

[9] 吕 瑞, 易 树华, 李 增军 (2015). 脾边缘区淋巴瘤91例临床疗效分析[J]. 中国实验血液学杂志.

[10] Casulo C, Byrtek M, Dawson KL (2015). Early relapse of follicular lymphoma after rituximab plus cyclophosphamide, doxorubicin, vincristine, and prednisone defines patients at high risk for death: an analysis from the national lymphocare study[J]. J Clin Oncol.

[11] 韩 倩楠, 胡 瑾, 陆 丰艺 (2024). POD24在霍奇金淋巴瘤中的预后意义[J]. 临床血液学杂志.

[12] 马 瑞雪, 张 芊芊, 陈 惠敏 (2024). 24个月内疾病进展在套细胞淋巴瘤中的预后意义[J]. 中国实验血液学杂志.

[13] Advani RH, Skrypets T, Civallero M (2021). Outcomes and prognostic factors in angioimmunoblastic T-cell lymphoma: final report from the international T-cell Project[J]. Blood.

[14] Tracy SI, Larson MC, Feldman AL (2019). The utility of prognostic indices, early events, and histological subtypes on predicting outcomes in non-follicular indolent B-cell lymphomas[J]. Am J Hematol.

[15] Iannitto E, Ambrosetti A, Ammatuna E (2004). Splenic marginal zone lymphoma with or without villous lymphocytes: hematologic findings and outcomes in a series of 57 patients[J]. Cancer.

[16] 吕 瑞, 阎 禹廷, 易 树华 (2020). POD24在106例伴骨髓侵犯脾边缘区淋巴瘤中的预后意义[J]. 中华血液学杂志.

[17] Nozaki K, Sugahara H, Ueda S (2020). Pretreatment serum soluble interleukin-2 receptor level predicts survival in patients with newly diagnosed follicular lymphoma[J]. Leuk Lymphoma.

[18] Yoshizato T, Nannya Y, Imai Y (2013). Clinical significance of serum-soluble interleukin-2 receptor in patients with follicular lymphoma[J]. Clin Lymphoma Myeloma Leuk.

[19] Camacho FI, Mollejo M, Mateo MS (2001). Progression to large B-cell lymphoma in splenic marginal zone lymphoma: a description of a series of 12 cases[J]. Am J Surg Pathol.

[20] Parry-Jones N, Matutes E, Gruszka-Westwood AM (2003). Prognostic features of splenic lymphoma with villous lymphocytes: a report on 129 patients[J]. Br J Haematol.

[21] Dungarwalla M, Appiah-Cubi S, Kulkarni S (2008). High-grade transformation in splenic marginal zone lymphoma with circulating villous lymphocytes: the site of transformation influences response to therapy and prognosis[J]. Br J Haematol.

[22] Freeman CL, Savage KJ, Villa D (2018). Frontline therapy with bendamustine and rituximab (BR) in follicular lymphoma: prognosis among patients with progression of disease by 24 months (POD24) is poor with majority having transformed lymphoma[J]. Blood.

